# Current status of enhanced recovery after surgery (ERAS) protocol in gastrointestinal surgery

**DOI:** 10.1007/s12032-018-1153-0

**Published:** 2018-05-09

**Authors:** Michał Pędziwiatr, Judene Mavrikis, Jan Witowski, Alexandros Adamos, Piotr Major, Michał Nowakowski, Andrzej Budzyński

**Affiliations:** 1 2nd Department of General Surgery, Jagiellonian University Medical College, Kopernika 21, 31-501 Krakow, Poland; 2 Centre for Research, Training and Innovation in Surgery (CERTAIN Surgery), Krakow, Poland; 3 Carol Davila University of Medicine and Pharmacy, Sector 1, Strada Dionisie Lupu 37, 030167 Bucharest, Romania; 4 Department of Medical Education, Jagiellonian University Medical College, św. Łazarza 16, 31-530 Krakow, Poland

**Keywords:** Enhanced recovery after surgery, Perioperative care, Laparoscopy, Complications, Length of stay

## Abstract

Enhanced Recovery After Surgery (ERAS) is an evidence-based paradigm shift in perioperative care, proven to lower both recovery time and postoperative complication rates. The role of ERAS in several surgical disciplines was reviewed. In colorectal surgery, ERAS protocol is currently well established as the best care. In gastric surgery, 2014 saw an establishment of ERAS protocol for gastrectomies with resulting meta-analysis showing ERAS effectiveness. ERAS has also been shown to be beneficial in liver surgery with many centers starting implementation. The advantages of ERAS in pancreatic surgery have been strongly established, but there is still a need for large-scale, multicenter randomized trials. Barriers to implementation were analyzed, with recent studies concluding that successful implementation requires a multidisciplinary team, a willingness to change and a clear understanding of the protocol. Additionally, the difficulty in accomplishing necessary compliance to all protocol items calls for new implementation strategies. ERAS success in different patient populations was analyzed, and it was found that in the elderly population, ERAS shortened the length of hospitalization and did not lead to a higher risk of postoperative complications or readmissions. ERAS utilization in the emergency setting is possible and effective; however, certain changes to the protocol may need to be adapted. Therefore, further research is needed. There remains insufficient evidence on whether ERAS actually improves patients’ course in the long term. However, since most centers started to implement ERAS protocol less than 5 years ago, more data are expected.

## Introduction

Enhanced Recovery After Surgery (ERAS) is an evidence-based multimodal perioperative protocol focused on stress reduction and the promotion of a return to function [1]. ERAS has been proven to lower both recovery time and postoperative complication rates while being cost-effective at the same time [2, 3]. It fundamentally shifts the traditional patient care in surgical wards to one that standardizes it based on published evidence [4]. Inspired by Danish Professor of surgery Henrik Kehlet, ERAS protocol questioned traditional perioperative care including: prolonged fasting, mobility limitations, mechanical bowel preparation, routine use of drains, and the slow return to eating normally postoperatively [4]. Kehlet theorized that the avoidance of such perioperative doctrine shortens the length of hospitalization by reducing the metabolic stress, fluid overload, and insulin resistance placed on the body [5]. Professors Kenneth Fearon and Olle Ljungqvist added postulates to the ERAS protocol, developing the ERAS study group in 2001 and the ERAS Society in 2010. The international ERAS study group consisted of surgeons and anesthesiologists who reviewed literature and evidence of the most optimal perioperative care [4]. They created an ERAS protocol of 20 items along with a database to support the implementation of these principles. The protocol divided the perioperative period, into pre-, intra-, and postoperative time periods based on the aggregation of marginal gains theory. This theory identifies, divides, and adapts each step taken through the entire perioperative patient journey to facilitate the efficient and safe progress from preoperative assessment to discharge and rehabilitation [6]. 2010 saw the establishment of the ERAS Society with the goal of an international network of regional and national expert centers that facilitated ERAS protocol utilization [5]. Currently, there is growing evidence that ERAS is beneficial in many other disciplines including colorectal, gastric, pancreatic, esophageal bariatric as well as in non-gastrointestinal specialties [7–10].

**Fig. 1:**
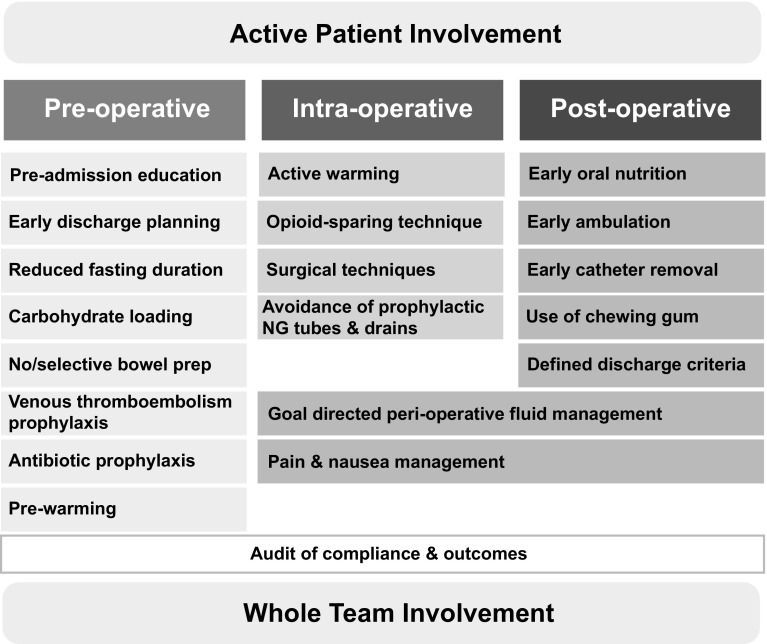
Key components of ERAS protocol

This care focuses on counseling preoperatively, optimizing nutrition, standardizing analgesia without opioid use, minimizing electrolyte and fluid imbalance, using the most minimally invasive approaches, and promoting early ambulation and feeding [5]. See Fig. 1 for overview of ERAS items.

## How does ERAS work?

The body physiologically responds to stress in a catabolic manner. The central nervous system mediates this, resulting in the production of various stress hormones and inflammatory mediators [5]. More importantly, insulin resistance develops. Unlike traditional care, ERAS aims to attenuate the development of insulin resistance, a key element in prolonged recovery and increased morbidity. The larger the operation, the greater the graded response of resistance. Despite the developing hyperglycemia, a reduction of muscle and fat glucose uptake occurs. The loss of lean body mass coupled with the reduced glucose uptake and storage in muscle leads to reduced muscle function. This impairs mobilization. Further, noninsulin-sensitive cells increase their glucose uptake. This increase can lead to several postoperative complications, such as infections and cardiovascular problems [11].

Beginning with preoperative counseling, clear information to patients before surgery decreases anxiety, facilitates postoperative recovery and pain control, and increases care plan adherence, allowing for earlier recovery and discharge [12]. ERAS protocol suggests against the previously standard mechanical bowel preparation (MBP), which has been proven to result in dehydration, along with fluid and electrolyte imbalances. MBP was meant to rid the large bowel of solid feces and lower the bacterial content; however, this practice in fact liquefies the feces which increases the risk of surgical spilling and does not reduce the number of bacterial organisms in the bowel [2]. Preoperative fasting has been a part of traditional surgery protocol to avoid pulmonary aspiration; however, no evidence supports this. Preoperative fasting instead exacerbates the already increased metabolic stress found postoperatively [13]. A metabolically fed state for surgery can be achieved by the ingestion of a clear carbohydrate-rich beverage before midnight and 2–3 h before surgery. This reduces preoperative thirst, hunger, anxiety, and postoperative insulin resistance [8]. The anabolic state that carbohydrate loading produces in the patient causes less postoperative nitrogen and protein losses and better maintenance of mass and muscle strength [2].

Meta-analyses have shown that low molecular weight heparin (LMWH) is equally as effective as low-dose subcutaneous unfractionated heparin in reducing the occurrence of deep vein thrombosis, pulmonary embolism, and overall mortality in patients. LMWH is preferable because of its once a day dosing and lower risk of heparin-induced thrombocytopenia [12]. Research shows the preemptive control of possible anaerobic and aerobic infections using prophylactic antibiotics is effective [14]. Studies support the fact that preservation of normal body temperature reduces wound infections, cardiac complications, bleeding, and transfusion requirements. This can be accomplished by forced air heating of the upper body, intravenous fluids given with extending heating to 2 h before and after surgery for additional benefits [13]. Traditional surgery protocol often included the dosing of IV fluids that outweighed the losses during surgery. By delaying the return of normal gastrointestinal functioning, impairing wound and anastomosis healing, and affecting tissue oxygenation, such regimes increased hospital stay. Evidence suggests that limiting postoperative IV sodium-rich fluid administration by stopping IV infusions and beginning early oral fluids, even on the first day postoperatively, can reduce hospital stay and postoperative complications such as ileus [12].

According to patient experiences, postoperative nausea and vomiting is more stressful than pain. The risk factors for these symptoms include female gender, non-smokers, history of motion sickness, and postoperative use of opioids. Individuals with at least 2 of these should be administered either dexamethasone sodium phosphate prophylaxis at the beginning or serotonin receptor antagonists at the end of the surgical procedure [13]. Drainage should not be used after uncomplicated procedures, as it does not lower the risk or severity of anastomotic leaks [14]. The use of minimally invasive surgical techniques has been shown to reduce complications, speed recovery, and lower pain. Nasogastric decompression should be avoided due to the occurrence of fever, atelectasis, and pneumonia [15]. Complete avoidance or at least removal of nasogastric tubes before the reversal of anesthesia is vital in reducing the risk of pneumonia while supporting the progression to intake of solids [13]. Long acting premedication, such as opioids, long acting sedatives, and hypnotics, can prolong recovery by delaying mobilization and the resumption of a normal diet. An earlier return to normal diet both supports mobilization, energy, and protein supply and reduces starvation-induced insulin resistance. The early removal of urinary catheters supports mobilization [14]. To reduce the risk of ileus, strategies include epidural analgesia in open surgery, avoidance of opioids and fluid overload, and oral laxatives usage early after surgery. Discharge should occur as soon as the patient has a solid food diet, bowel movements, orally controlled pain, sufficient mobility for self-care, and no complications requiring hospital care [12]. What is probably the most important in ERAS—its aim is not to discharge a patient from hospital as soon as possible. It rather aims to prepare him for early discharge by making him fully capable of going home.

## ERAS in different surgical disciplines

The use of ERAS has been most extensively studied in colorectal surgery. A multicenter randomized LAFA trial, the paramount Dutch study, compared four groups of patients undergoing open/laparoscopic surgery with/without ERAS [16]. It was shown that a combination of ERAS and laparoscopy was associated with significant improvements in postoperative recovery. Next RCTs and several subsequent meta-analyses clearly showed that the introduction of ERAS to colorectal surgery decreased postoperative morbidity by 40–50% (mainly non-surgical) and shortened LOS by 2–3 days [17–19]. Therefore, Greco et al. concluded that new RCTs were not required to compare ERAS with the standard of care in colorectal surgery. Rather, it is apparent from current evidence that new policies are needed to help implement ERAS protocol worldwide [17]. Moreover, it has been demonstrated that a combination of ERAS and laparoscopy helps eliminate some well-established risk factors for prolonged LOS and complications [20, 21]. Importantly, ERAS can be successfully implemented in both colonic and rectal resections providing similar outcomes and level of adherence to the protocol, even in patients with advanced cancer [22, 23]. The position of ERAS protocol in colorectal surgery is nowadays well established as the best care and it is very unlikely that future trials will change this.

While proposed for gastric surgeries, ERAS protocol implementation is still being studied [24–26]. In 2014, Yu et. al’s meta-analysis of 400 patients showed that postoperative hospital stay, time to first flatus, and hospital costs were significantly reduced in patients who received ERAS perioperative care [27]. Additionally in 2014, an international committee within the ERAS society assembled an evidence-based 25-item long protocol for those patients undergoing gastrectomies [28]. A 2015 meta-analysis, including 7 RCTs and 524 patients, showed that ERAS treatment was associated with shorter postoperative hospitalization, less hospitalization expenditure, less pain, and better quality of life [29]. Then in 2018, a subsequent meta-analysis similarly showed ERAS led to shortened time to first flatus, postoperative hospital stay, postoperative CRP levels, and hospitalization fees. Due to the limitations of the study, however, further larger and multicenter studies are warranted to validate the findings [30]. In particular, the use of drains in gastric surgery, not in compliance with ERAS protocol, had been debated in the past. However, a Cochrane review of total or subtotal gastrectomies performed between 1996 and 2014, showed no evidence in support of drainage regarding morbidity-mortality, nor in the diagnosis or management of leakage [31]. Lastly, early postoperative oral feeding as compared with traditional, or late, feeding is associated with shorter hospital length of stay and is not associated with an increase in clinically relevant complications [32].

ERAS has also been shown to be beneficial in liver surgery and its implementation has started in many centers [33]. Some reports show that current practices in hepatic surgery already cover several items of the modern perioperative care protocols, as suggested in a 2014 study by Wong-Lun-Hing et al [34]. However, this needs further optimization, standardization, and broader research. A step towards this standardization was the publication of the ERAS Society Recommendations in 2016 [35]. Additionally, it is important to note a growing number of recently published trials, including randomized prospective studies, that confirm there is a place for ERAS in this surgical discipline. Although mentioned trials do not have an overwhelming number of subjects (62 patients in Kapritsou et al. study [36]; 160 in Qi et al. RCT [37]), they provide strong evidence of clinical safety and efficacy, even in major resections.

The advantages of ERAS in pancreatic surgery have been strongly established through a number of research papers, including both meta-analyses and guidelines; for example, a study on ERAS care post pancreaticoduodenectomy was published in 2012 [38]. Literature reviews agree that ERAS may be introduced without compromising patients’ safety, although there is still a need for large-scale, multicenter randomized trials [39, 40]. It seems one of the greatest concerns arises around minimally invasive pancreatic surgery because the evidence for its safety in cancer patients is still limited [41, 42]. As with hepatic surgery, recent high-quality trials have provided new evidence in regard to the implementation of ERAS in pancreatic surgery. For instance, results of Takagi et al. RCT published in January 2018 showed not only significantly lower rates of complications and readmissions, but also improved patients’ quality of life when treated with ERAS [43]. On the other hand, one has to bear in mind that pancreatic surgery is particularly prone to specific complications such as delayed gastric emptying or pancreatic fistula formation which can severely affect both LOS and postoperative compliance with early enteral feeding.

## Difficulties in ERAS implementation

A large body of evidence demonstrates the success rates of ERAS protocol, showing decreased recovery times, shortened hospital stays, reduced hospitals costs, and increased patient satisfaction. However, ERAS’s challenge to traditional surgical doctrine has led to slow implementation [44]. Every member of the team must overcome the resistance to change and embrace ERAS protocol [45]. Resistance to change, however, is just one of the many barriers. Additionally, compliance to all protocol items is crucial and often difficult to accomplish. One single center study proved that a 50–90% increase in the compliance rate decreased complication rates by 20% and the length of stay by 4 days [4]. Similarly, another single center study demonstrated that a compliance rate of at least 80% is needed to decrease the length of hospital stay, and, that this compliance rate takes approximately 6 months and the treatment of 30 patients to successfully achieve [46]. In 2015, The ERAS Compliance Group showed in a large-scale study on over 1500 colorectal cancer patients that increasing ERAS compliance correlates with fewer complications [47]. This trend was later confirmed by two studies observing patients undergoing laparoscopic surgery for colorectal cancer. The first study showed that the decrease in both the rate of complications and length of hospital stay was correlated with level of compliance to ERAS protocol; there was no correlation to patient specific comorbidities or stage of cancer. The second study further supported the validity of this trend, when it demonstrated a significant decrease in complication rates with increasing compliance (35.7% vs. 36.4% vs. 16.4%, *p* = 0.0024) as well as a decrease in the severity of complications that did occur [20, 48].

This correlation between compliance and clinical outcomes raises the issue on how to maximize patients’ adherence to the protocol. Some authors suggest auditing patient compliance weekly, potentially allowing for the implementation of any necessary changes to the protocol [5, 49]. It is also important to educate patients [50]. With no doubt, the early implementation period is the most inconsistent when it comes to ERAS compliance. According to Pędziwiatr et al. a multidisciplinary team needs at least 40 cases and 6 months to reach satisfactory level of adherence to the protocol [46].

Still, reports from institutes that use ERAS in perioperative care are optimistic. Compliance rate is usually above 60% and can be as high as over 90% [20, 51, 52]. Even in groups with lower compliance (< 70%), implementation of all ERAS items is beneficial and improves short-term outcomes [48]. The question is whether a high level of compliance can be sustained in long-term observation. Roulin et al. found that over the 8-month study period, reasons for non-compliance are usually (in almost 80% of cases) medically justified and that they are mostly observed in the postoperative period [53].

In another study, all members of the ERAS multidisciplinary team from nurses to surgeons were interviewed to better understand the barriers and enablers of ERAS. When asked what the largest hindrance is to successfully implementing ERAS, some responded institutional barriers, such as a lack of nursing staff and financial resources. Another group blamed the lack of communication and collaboration within the team [44].

Recent studies claim that the successful implementation of ERAS protocol requires a multidisciplinary team coupled with a willingness to change and a clear understanding of how to utilize the protocol [5]. In their study on the barriers of ERAS utilization, Kahokehr et. al recommended that the keys to successful implementation were developing a multidisciplinary team, distributing patient educational materials, and modifying the postoperative ward into a patient friendly rehabilitation center. A prospective study on 425 patients treated under ERAS guidelines in the Netherlands, Norway, Sweden, United Kingdom, and Denmark showed that the lowest compliance rates occurred postoperatively. The author called for patients to complete their own daily logs and the reeducation of the members of the team to clarify their individual roles as well as the multidisciplinary protocol [54]. An additional study of 107 patients treated with ERAS guidelines in the Netherlands called for continuous education to ensure compliance [55]. Kisielewski et al. found that Polish surgeons followed several ERAS elements such as antibiotic and antithrombotic prophylaxis, postoperative oxygen therapy, and lack of nasogastric tubes. On the other hand, several elements were not followed. Surgeons were not willing to change their practice but were supportive of changes in anesthesiologist-dependent elements of perioperative care that did not interfere with their own work, such as restrictive fluid therapy and the use of transversus abdominis plane blocks [56].

## ERAS in specific patient populations

Regarding the elderly population, a systematic review of 16 studies including 5965 patients supported the safety of ERAS in the elderly, with similar prevalence of morbidity and mortality compared to a younger population [57]. Baek et al. found no difference in postoperative results between patients below and above 70 years of age following enhanced recovery protocol [58]. However, ERAS protocol requires active participation and adherence to it within the elderly population had yet to be studied. A study of ERAS protocol adherence compared 513 patients: 311 patients in the younger group and 202 in the older group [59]. The overall adherence to ERAS protocol had a median of 78% (67–85%) in younger and 74% (64–85%) in older patients. Adherence was 100% (83–100%) versus 100% (83–100%) for preoperative protocol, 80% (80–85%) versus 80% (75–100%) for intra-operative protocol, and 72% (76–81%) versus 69% (52–81%) for postoperative protocol. No significant differences were noted for any of the three phases, despite the older population having significantly more comorbidities, worse disability scores and more emergency procedures. One difference in the studied groups was that urinary catheters and nasogastric tubes were retained longer in the elderly population. However, no differences in urinary retention or postoperative ileus were observed [59]. Kisialeuski et al. further supported ERAS implementation in the elderly; the authors demonstrated again that even with higher ASA grades, ERAS shortened the length of hospitalization and did not lead to a higher risk of postoperative complications or readmissions [60]. Although there are relatively many studies in literature comparing younger patients with the elderly, there are discrepancies in the age cutoffs used in these studies. For example, Wang et al. [61], Bagnall et al. [57], and Kisialeuski et al. defined the cutoff as over 65 years [60], Baek et al. [58] and Slieker et al. [59] defined the cutoff as over 70 years old, and even further Verheijen et al defined the cutoff at 80 years old [62].

Postoperative complications and prolonged hospital stays remains a problem in emergency surgeries. For obvious reasons, not all ERAS items are possible to implement in the emergency setting (e.g., preoperative carbohydrate loading in mechanical bowel obstruction or limited feasibility of minimally invasive surgery, needs for drains etc). Lohsiriwat et al. investigated the feasibility of implementing ERAS protocol in the setting of emergent colorectal surgery. He compared the surgical outcomes of patients treated with ERAS protocol with those receiving conventional postoperative care in a matched case-control study. A reduction in hospital stay, time to first flatus, and time to resume normal diet was found in those receiving ERAS based care, without an increase in 30-day readmission or postoperative complications. He concluded that implementation of selected ERAS items in the setting of emergency colorectal surgeries was feasible and effective. Limitations in this study included its small sample size and selective inclusion of low risk patients [63]. Gonenc et al. demonstrated safe usage of ERAS guidelines in certain gastrointestinal emergent surgeries. When comparing 47 patients undergoing emergency surgery for perforated peptic ulcers, treatment with ERAS protocol effectively decreased the length of hospital stay [64]. Wisely et al. investigated the utilization of ERAS protocol in 370 patients undergoing emergent major abdominal surgery. The ERAS patient group had significantly reduced presence of catheters, drains, patient-controlled analgesia, urinary tract infections, urinary retention, and chest infections. While the results supported ERAS implementation in emergency abdominal surgeries, only some of the ERAS guidelines were implemented and further research is needed [65]. This call for further trials was supported by Paduraru et. al’s systematic review of the successful implementation and surgical outcome of ERAS protocol for emergency surgeries. The authors showed that the number of employed ERAS items ranged from 11 to 18 of the 20 recommended by the ERAS Society for elective procedures; patients treated within the guidelines had fewer postoperative complications, shorter hospital stays, with equal or lower mortality rates in certain studies. It seems that ERAS utilization in emergency setting is possible and effective; however, certain changes to the protocol may need to be adapted. Therefore, further research is needed to fully establish the role of ERAS in decreasing major morbidity and mortality [66].

## ERAS impact on long-term outcomes

There is still very little evidence on how ERAS implementation benefits patients long term [5]. Reports have been published which suggest that enhanced recovery protocols can increase long-term survival; however, these results are quite recent and need to be studied further [67]. One of these reports, performed by Curtis et al., compared laparoscopy and open approach surgeries with ERAS protocol implementation in all patients; these additional variables made the results more controversial and less clear [59]. However, a Gustafsson et al. study has more clear conclusions. Gustafsson et al. analyzed 5-year survival in cohorts with different adherences [68]. Patients who had higher compliance (≥ 70%) to the protocol had reduced risk of 5-year cancer-specific death, HR 0.58 (95% CI 0.39–0.88). Restricted perioperative fluid therapy, one of the elements of ERAS protocol, has also been shown to be related to improved 5-year survival (cancer-specific death, HR 0.45, 95% CI 0.25–0.81) [69].

These results still do not provide enough sufficient evidence to be decisive on whether ERAS does actually improve patients course in the long term. We should expect more results in upcoming years, since most centers started to comprehend and implement enhanced recovery protocol less than 5 years ago.

## Conclusions

Interest in the implementation of modern perioperative care pathways based on ERAS principles results from growing evidence that it is safe, feasible, and associated with improved outcomes. ERAS reduces complications, shortens LOS, and thus leads to economic benefits in the majority of surgical disciplines. However, there are still challenges in sustaining a high level of compliance with ERAS items in the long term as well as the introduction of ERAS to emergency surgery. It shows clearly that changing surgical dogmas is more difficult that one could assume. Therefore, new implementation strategies are needed in order to increase the popularity and utilization of this approach.
